# Photocatalysis and Photoelectrochemical Properties of Tungsten Trioxide Nanostructured Films

**DOI:** 10.1155/2014/843587

**Published:** 2014-03-20

**Authors:** Chin Wei Lai

**Affiliations:** Nanotechnology & Catalysis Research Centre (NANOCAT), Institute of Postgraduate Studies (IPS), University of Malaya, 3rd Floor, Block A, 50603 Kuala Lumpur, Malaysia

## Abstract

Tungsten trioxide (WO_3_) possesses a small band gap energy of 2.4–2.8 eV and is responsive to both ultraviolet and visible light irradiation including strong absorption of the solar spectrum and stable physicochemical properties. Thus, controlled growth of one-dimensional (1D) WO_3_ nanotubular structures with desired length, diameter, and wall thickness has gained significant interest. In the present study, 1D WO_3_ nanotubes were successfully synthesized via electrochemical anodization of tungsten (W) foil in an electrolyte composed of 1 M of sodium sulphate (Na_2_SO_4_) and ammonium fluoride (NH_4_F). The influence of NH_4_F content on the formation mechanism of anodic WO_3_ nanotubular structure was investigated in detail. An optimization of fluoride ions played a critical role in controlling the chemical dissolution reaction in the interface of W/WO_3_. Based on the results obtained, a minimum of 0.7 wt% of NH_4_F content was required for completing transformation from W foil to WO_3_ nanotubular structure with an average diameter of 85 nm and length of 250 nm within 15 min of anodization time. In this case, high aspect ratio of WO_3_ nanotubular structure is preferred because larger active surface area will be provided for better photocatalytic and photoelectrochemical (PEC) reactions.

## 1. Introduction

Design and controlled growth of nanostructure semiconductor assemblies has gained significant attention in recent years due to the scientific interests and potential applications [1, 2]. In this manner, WO_3_ is one of the famous electrochromic inorganic materials since Deb's discovery in 1969 [3]. WO_3_ film exhibits a broad range of functional properties, such as small band gap energy (2.4–2.8 eV), deeper valence band (+3.1 eV), stable physicochemical properties, and strong photocorrosion stability in aqueous solution [4–10]. The characteristics of WO_3_ film make them suitable for electrochromic layers in a smart window [11]. Many studies pertaining to WO_3_ nanostructures are mainly aimed at the formation of high active surface area in view of their use in electrochromic applications [9, 10]. However, several studies have reported that growth of well-aligned and uniformity of anodic WO_3_ nanotubular structure was a difficult task and most of the studies were only able to grow anodic WO_3_ into nanoporous instead of nanotubular structure [7–10, 12, 13]. In the present study, we describe the synthesis of well-aligned anodic WO_3_ nanotubes using electrochemical anodization technique in a fluorinated-based electrolyte. To the best of our knowledge, literatures on optimization of the geometrical features of regular anodic WO_3_ nanotubular structures are still lacking. Thus, such mechanistic studies and understanding are very important to tailor the desired length, pore size, and wall thickness of ordered WO_3_ nanotubular structures for high surface area to volume ratio. In this study, a comprehensive experiment was conducted to control the one-dimensional nanostructure of anodic WO_3_ using electrochemical anodization to achieve effective photocatalytic degradation of MO dye and H_2_ gas generation via PEC water splitting process.

## 2. Experimental Procedure

The high purity (99.95% purity with 0.1 mm in thickness) tungsten (W) foils from Alfa Aesar USA were used in this study. Prior to anodization, W foils were degreased in ultrasonic bath containing ethanol for 30 minutes. The foils were then rinsed in deionized water and dried in nitrogen stream. Then, anodization was performed in a two-electrode configuration bath with W foil served as the anode and the platinum electrode served as the counter electrode. The electrolyte is composed of 100 mL of 1 M of sodium sulfate (Na_2_SO_4_, Merck, USA) solution with ammonium fluoride (NH_4_F, Merck, USA) at 40 V with sweep rate of 1 V/s. In the present study, different content of NH_4_F (0.3, 0.5, and 0.7 wt%) will be added into 1 M Na_2_SO_4_ solution for several anodization duration (15, 30, and 60 min) in order to investigate the formation of anodic WO_3_ nanotubular structures. As-anodized anodic WO_3_ samples were cleaned using acetone (J.T. Baker, Nederland) and dried in nitrogen stream after anodization process. The morphologies of anodic WO_3_ nanostructures were observed by field emission scanning electron microscopy (FESEM), using a FEI Quanta 200 (FESEM model, USA) at a working distance of around 1 mm. The cross-sectional observation was carried out on mechanically bent samples to get the thickness of the oxide layer. The chemical stoichiometry of the sample was characterized using energy dispersive X-ray (EDX) analysis, which is equipped in the FESEM. In order to assess the photocatalytic performance of the anodic WO_3_ nanostructure formed, anodized W foil of 25 mm × 25 mm was prepared and placed in 200 mL of 30 ppm MO dye in a customized photoreactor made of quartz glass. Two different surface morphologies of anodic WO_3_ nanostructure were selected for MO dye degradation purpose (e.g., oxide layer and nanotubular structure). In the present study, both samples were left in the photoreactor for 30 min in a dark environment to achieve adsorption/desorption equilibrium. Then, both samples were photo-irradiated at room temperature by using a 150 W Xenon solar simulator (Zolix LSP-X150) with intensity of 800 W/m^2^. A 5 mL solution was removed at an interval of 1 h from the photoreactor, and concentration of the solution was measured using a UV spectrophotometer (PerkinElmer Lambda 35). Next, the photoelectrochemical properties of the selected samples were further characterized using a three-electrode water splitting cell, with WO_3_ nanotubes as the working electrode, platinum rod as the counter electrode, and saturated calomel electrode (SCE) as the reference electrode. The bath with electrolyte composed of 0.5 M sulfuric acid aqueous solution was selected in this experimental work. The H_2_ gas generated at platinum rod was collected using the water displacement technique. As the H_2_ gas was produced in counter electrode in water splitting chamber, it was bubbling up into inverted burette. The volume of H_2_ gas was determined by reading the gas level on the side of burette.

## 3. Results and Discussion

**Figure 1:**
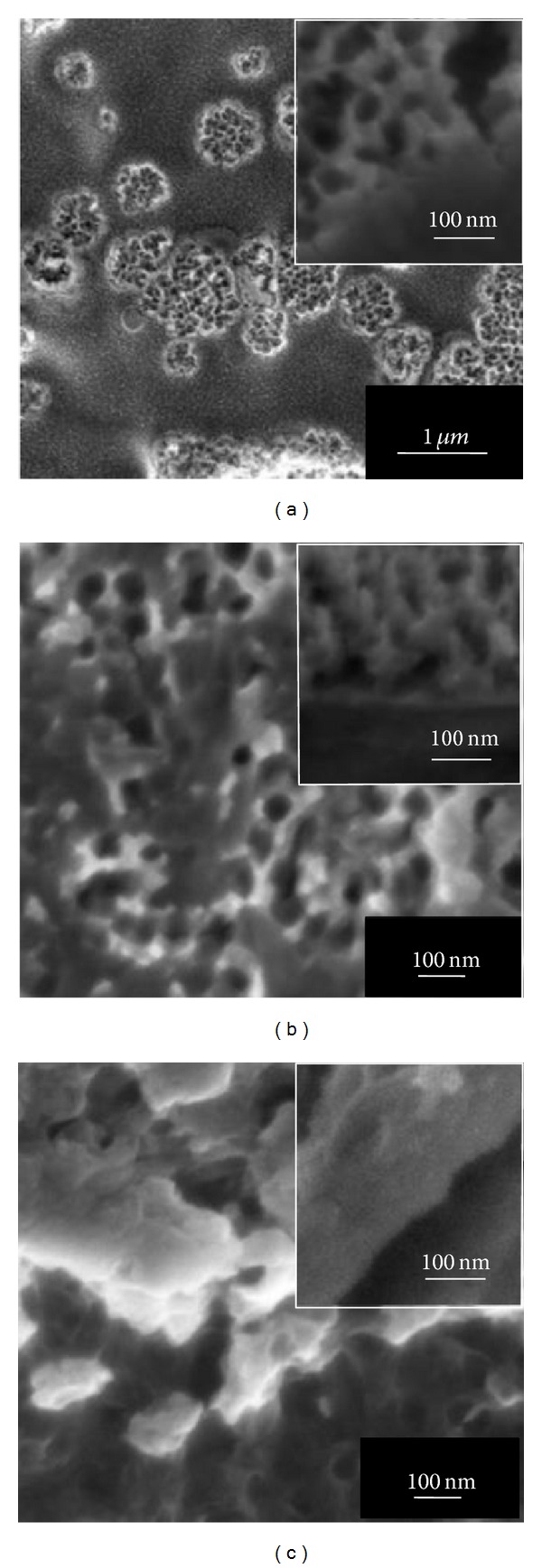
FESEM images of anodic WO_3_ nanostructures obtained in 1 M Na_2_SO_4_ electrolyte containing 0.3 wt% NH_4_F for different anodization times at 40 V: (a) 15 min, (b) 30 min, and (c) 60 min. Insets show the cross-sectional view of anodic oxide layers.

**Figure 2:**
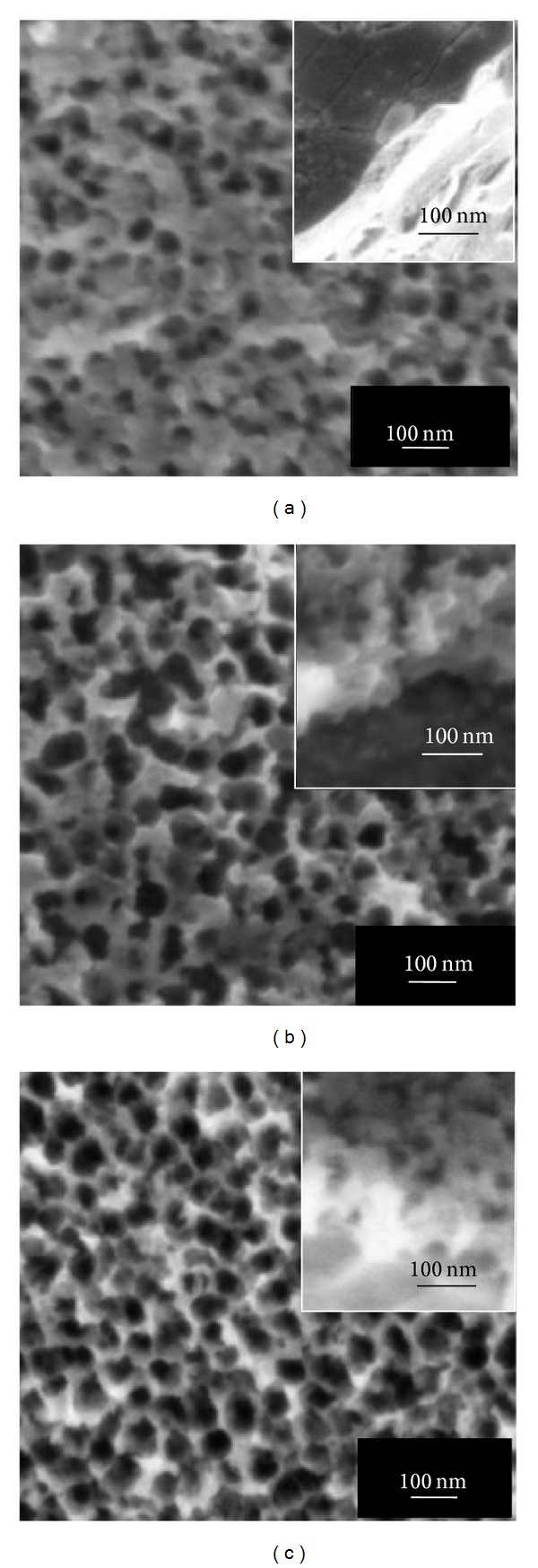
FESEM images of anodic WO_3_ nanostructures obtained in 1 M Na_2_SO_4_ electrolyte containing 0.5 wt% NH_4_F for different anodization times at 40 V: (a) 15 min, (b) 30 min, and (c) 60 min. Insets show the cross-sectional view of anodic oxide layers.

**Figure 3:**
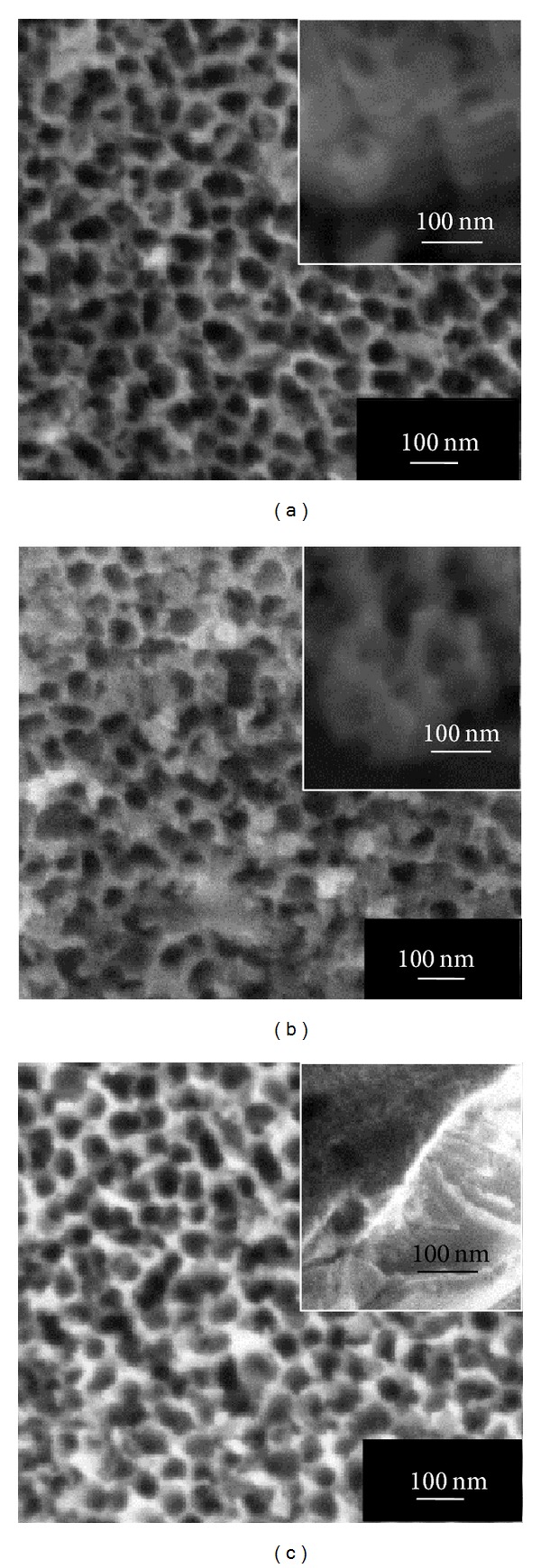
FESEM images of anodic WO_3_ nanostructures obtained in 1 M Na_2_SO_4_ electrolyte containing 0.7 wt% NH_4_F for different anodization times at 40 V: (a) 15 min, (b) 30 min, and (c) 60 min. Insets show the cross-sectional view of anodic oxide layers.

**Figure 4:**
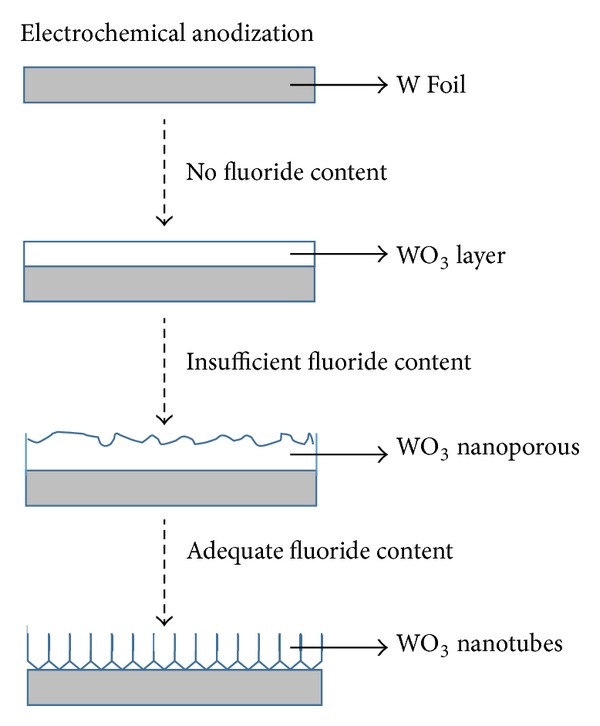
Schematic illustration of formation and mechanistic studies of anodic WO_3_ nanostructured film with and without fluoride content during electrochemical anodization stage.

In the present study, the effect of fluoride content and anodization time on the morphology of anodic WO_3_ layer was investigated.  Figure 1 to Figure 3 showed the surface morphologies of anodic WO_3_ layer in different fluoride content electrolyte from 15 min up to 60 min of anodization time. As shown from the FESEM images, the appearance of anodic WO_3_ layer was strongly dependent on the fluoride content and anodization times. Then, the EDX analysis was employed to investigate the composition of element of W and O from the anodic WO_3_ layer. Based on the results obtained, the atomic percentage of W element was about 55 at% and O element was about 45% at%. During electrochemical anodization, fluoride content played an important role in controlling the chemical dissolution rate at the interface of W/WO_3_ [14].  Figure 1 exhibited variations of WO_3_ surface morphology under low 0.3 wt% fluoride content electrolyte for different anodization times. It could be observed that formation of oxide layer on W foil was incomplete at 15 min of anodization time (Figure 1(a)). Interestingly, only thin compact oxide layer with randomly pits was formed after prolonging the anodization time to 30 min and 60 min (Figures 1(b) and 1(c)). The resultant thickness of oxide layer was approximately 100 nm. These results indicated that low fluoride concentration was insufficient in forming the deep and large pore size on the oxide layer due to the inactive chemical dissolution reaction [15]. In this case, oxygen ions within the electrolyte through the W surface towards the W/WO_3_ interface induce further growth of the oxide layer under applied potential. The high electric field across the oxide layer of WO_3_ and subsequently induce the polarization of W–O bonding, which is able to transfer the W^6+^ ions from the pores and leave behind random pits [14, 15]. By further increasing the content of fluoride to 0.5 wt%, the irregular anodic WO_3_ nanoporous structure with thickness of approximately 200 nm could be observed from Figures 2(a) to 2(c). However, it could be noticed that the pore diameter on the oxide layer was increasing up to 70 nm when prolonging the anodization time to 60 min. The uniform anodic nanoporous WO_3_ layer could be achieved in 1 M Na_2_SO_4_ electrolyte composed of 0.5 wt% fluorides content. When the fluoride content was further increased to 0.7 wt%, a hollow cylinder oxide nanostructure was observed as shown in Figure 3(a), which indicated that the amount of fluoride in the electrolyte was sufficient to increase the chemical dissolution rate. This condition led to further acidification reactions to develop the nanoporous structure into nanotubular structure [16–18]. In this case, tungsten fluoro-complex ions within the electrolyte playing an important role in inducing chemical dissolution to enlarge and deepen pores and eventually transforming to nanotubular structure [14]. It is noteworthy to mention that the WO_3_ nanotubes with diameters of approximately 85 nm and lengths of 250 nm were successfully formed when the fluoride content was increased to 0.7 wt%. However, nanotubular structure disappeared when further the anodization time increased to 30 min and 60 min and eventually resulted in irregular nanoporous structure (Figures 3(b) and 3(c)). The reason might be attributed to the excessive chemical etching on the wall surface of nanotubes during the chemical dissolution reactions. Thus, optimization of fluoride content identified in our electrolyte was 0.5 wt% in order to grow the well-aligned one-dimensional WO_3_ nanotubes for 15 min electrochemical anodization duration.  Figure 4 presented a simple schematic illustration of formation of anodic WO_3_ nanostructured film during electrochemical anodization stage in the presence of insufficient and adequate fluoride content.

**Figure 5:**
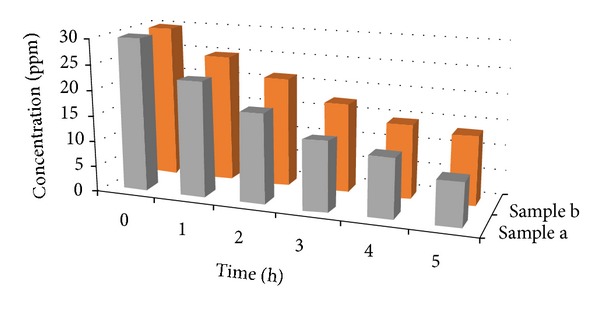
Photodegradation of MO dye: (a) anodic WO_3_ nanostructures obtained in 1 M Na_2_SO_4_ electrolyte containing 0.7 wt% NH_4_F for 15 min at 40 V, (b) anodic WO_3_ nanostructures obtained in 1 M Na_2_SO_4_ electrolyte containing 0.3 wt% NH_4_F for 15 min at 40 V.

**Figure 6:**
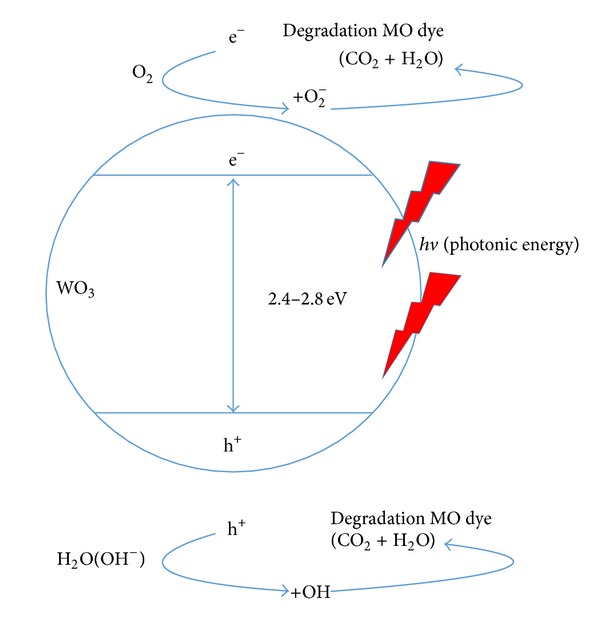
The overall mechanism of the photocatalytic degradation of MO dye using WO_3_ nanostructured film under solar illumination.

The photocatalytic removal ability of the selected sample of anodic WO_3_ oxide layer was compared with that of the anodic WO_3_ nanotubular structure by exposing the samples to MO dye under solar illumination. The initial MO dye concentration in the solution was fixed at 30 ppm. The changes in MO dye concentration were investigated within 5 hours and the result was shown in Figure 5. The degradation rates of MO dye concentration for sample “anodic WO_3_ oxide layer” and sample “anodic WO_3_ nanotubular structure” was decreased from 30 ppm to 13.5 ppm and 8.5 ppm, respectively. When anodic WO_3_ nanotubular structure exposed to the* hv* illumination that solar photonic energy is higher than its band gap energy (2.4–2.8 eV), the anodic WO_3_ itself will generate pairs of photo-induced electrons (e^−^) and holes (h^+^). In this manner, the e^−^ and oxygen molecule (O_2_) will combine to form super oxide anion (O_2_^•−^), whereas the h^+^ of anodic WO_3_ and water molecule (H_2_O) will generate hydroxyl radical (^•^OH). These powerful oxidizing agents (^•^OH and O_2_^•−^) will then decompose the MO dye (organic dye) into CO_2_ and H_2_O. This cycle will continue when the* hv* illumination is available. A simple schematic illustration of basic principal in photocatalytic degradation of MO dye is shown in Figure 6. In theoretical perspectives, the photocatalytic degradation performance of anodic WO_3_ can be related on the ability to generate pairs of charge carriers, which will release powerful oxidizing agents (^•^OH and O_2_^•−^) that are able to undergo the secondary reactions. In other words, anodic WO_3_ nanotubular structure with larger surface area of active reaction sites (inner and outer wall surface of nanotubes) has better photon absorption under* hv* illumination. The distance of light scattering inside the nanotubes extends and provides more photon absorption to trigger the photocatalytic degradation reaction.

**Figure 7:**
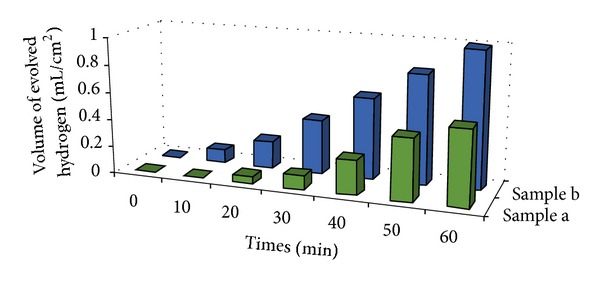
H_2_ evolution under solar illumination of (a) anodic WO_3_ nanostructures obtained in 1 M Na_2_SO_4 _electrolyte containing 0.3 wt% NH_4_F for 15 min at 40 V and (b) anodic WO_3_ nanostructures obtained in 1 M Na_2_SO_4_ electrolyte containing 0.7 wt% NH_4_F for 15 min at 40 V.

**Figure 8:**
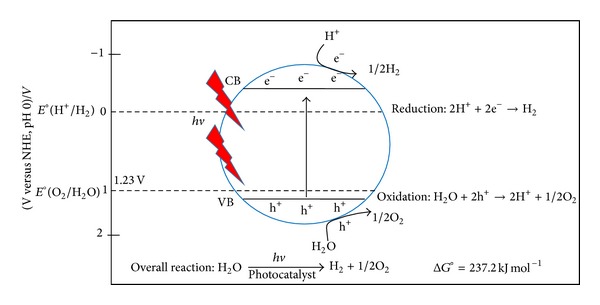
Basic principle of the overall water splitting for H_2_ generation using a WO_3_ nanostructured film.

On the other hand, the evolution rate of H_2_ gas generated from the photoelectrochemical (PEC) water-splitting process under solar illumination was measured. The H_2_ evolution as a function of time is shown in Figure 7. H_2_ generation rate from water splitting reaction increased linearly with increasing exposure time. The sample (anodic WO_3_ nanotubular structure) achieved a maximum evolution of approximately 1 mL/cm^2^ within 1 hour, which is relatively higher compared with the anodic WO_3_ oxide layer. The H_2_ production completely stopped after the termination of* hv* illumination. This observation clearly shows that H_2_ is only produced photocatalytically. A constant production rate of H_2_ gas could be observed in the present study. In theoretical perspectives, PEC water splitting process is the general term for a chemical reaction in which water is separated into O_2_ and H_2_ using anodic WO_3_ film that catalyze the water splitting reaction. A basic schematic diagram of such overall water splitting reaction using a semiconductor photocatalyst is presented in Figure 8. The water splitting reaction can be summarized as follows: 2H_2_O(l) → O_2_(g) + 2H_2_(g). The overall water splitting reaction is considered as a thermodynamically uphill reaction with a large Gibbs free energy of ΔG^0^ = +237.2 KJ mol^−1^. This reaction indicates that photon energy is required to overcome the large positive change in Gibbs free energy through PEC water splitting process [19, 20]. The light-driven water splitting process is triggered when anodic WO_3_ film absorbs photons from* hv* illumination with energies greater than its band gap energy. This light absorption generates negative e^−^ in the conduction band and positive h^+^ in the valence band. The h^+^ performs work at the anodic WO_3_ electrolyte interface oxidizing water molecules to create O_2_ and H^+^ ions within the electrolyte. Then, the e^−^ will move through the external circuit to the platinum electrode (counter electrode) where they reduce H^+^ ions creating H_2_ molecules due to the electric field or under external bias [20]. The PEC water splitting performance is consistent with the photocatalytic degradation. In summary, the self-organized WO_3_ nanotubular structure has strong ability to release much more photo-induced e^−^/h^+^ pairs than that of compact layer structure. Thus, it is crucial to maximize the active surface area of photocatalyst (anodic WO_3_) for better photocatalytic and photoelectrochemical performance.

## 4. Conclusion

In conclusion, complete transformation of W foil to one-dimensional WO_3_ nanotubes with an average diameter of 85 nm and length of 250 nm could be achieved within 15 min in an electrolyte composed of 1 M of Na_2_SO_4_ and 0.7 wt% of NH_4_F. The main reason attributed to the sufficiency of tungsten fluoro-complex ions induced chemical dissolution to enlarge and deepen pores and eventually transform to nanotubes. The ability to grow large active surface area of anodic WO_3_ nanostructures demonstrated a substantial enhancement in the degradation of MO dye and H_2_ generation via water splitting process, as compared to the anodic WO_3_ oxide layers.
